# Assessing the Co-Variates of Suicide-Related Ideation and Self-Harm in an Older Adult Population Attending Emergency Departments in Ireland

**DOI:** 10.1192/bjo.2024.163

**Published:** 2024-08-01

**Authors:** Fiona Hoare, Ann O'Donoghue, Colm Sweeney, Geraldine McCarthy, Anne Doherty

**Affiliations:** 1 St Vincent's University Hospital, Dublin 4, Ireland; 2 St Patrick's University Hospital, Dublin, Ireland; 3 National Forensic Mental Health Services, Dublin, Ireland; 4 University of Galway, Galway, Ireland; 5 Mater Misericordiae University Hospital, Dublin, Ireland; 6 UCD, Dublin, Ireland

## Abstract

**Aims:**

Older people (people aged 65 and older) have high rates of suicide, and self-harm is a major risk factor for suicide. While rates of self-harm decrease with age, rates of suicide increase amongst this age group. The aim of this project is to analyse data collected by the National Clinical Programme for Self-Harm and Suicide-related Ideation (NCPSHI) to identify real-life evidence of the characteristics associated with older people who present with self-harm and suicidal ideation to emergency departments (ED) in Ireland. In examining the variables associated with self-harm we may be better able to identify the characteristics of older adults who are at highest risk, including those presenting with high lethality attempts.

**Methods:**

The NCPSHI collects data on all patients who present with self-harm or suicide-related ideation to EDs in Ireland. We utilised a six-year cohort of anonymised data from the NCPSHI from 2018–2023, representing 5,041 presentations of older people (aged 60 and over); 6.9% of all presentations with self-harm and suicide-related ideation. We examined sociodemographic variables including sex, ethnic background, type of self-harm, lethality of self-harm and substance use contributing to the presentation, in addition to service use variables.

**Results:**

Older people were less likely to present with self-harm – 45% of older people vs 52% of adults under 60 (p < 0.001). However, those episodes of self-harm were more likely to be categorised as “high lethality” – 20% vs 12% of people under 60 (p < 0.001). Older people were also more likely to have a mental health admission – 25% vs 16% (p < 0.001). Older people were much less likely to present with substance misuse: 30% vs 45% (p < 0.001). There were also significant differences in methods of self-harm. Older people were more likely to attempt drowning (1.5% vs 1.1%) or overdose (21% vs 20%). This was the common method of self-harm across all age groups.

**Conclusion:**

Our results demonstrate the significant differences in characteristics of older people presenting to Irish emergency departments with self-harm versus younger people, where previously a paucity of data existed. The high lethality of self-harm amongst older people makes it imperative to identify the characteristics of self-harm in this population to understand the factors associated with increased risk and help us to develop treatments and services to meet their needs It also highlights the importance of providing education to staff working with this cohort to appropriately stratify and manage risk.

